# Behavioral correlates of the decision process in a dynamic environment: post-choice latencies reflect relative value and choice evaluation

**DOI:** 10.3389/fnbeh.2015.00261

**Published:** 2015-09-29

**Authors:** Justine Fam, Fred Westbrook, Ehsan Arabzadeh

**Affiliations:** ^1^School of Psychology, University of New South WalesSydney, NSW, Australia; ^2^John Curtin School of Medical Research, Eccles Institute of Neuroscience, Australian National UniversityCanberra, ACT, Australia; ^3^ARC Centre for Excellence for Integrative Brain Function, Australian National UniversityCanberra, ACT, Australia

**Keywords:** discrete-trial, choice, behavioral contrast, response latencies

## Abstract

One characteristic of natural environments is that outcomes vary across time. Animals need to adapt to these environmental changes and adjust their choices accordingly. In this experiment, we investigated the sensitivity with which rats could detect, and adapt to, multiple changes in the environment. Rats chose between two spouts which delivered 5% sucrose rewards with distinct probabilities. Across three phases, reward probabilities changed in size (large or small) and direction (increase or decrease). A discrete trial-structure was used, which allowed the choice process to be decomposed into three distinct response latency measures (choice execution latency, spout sampling duration, and trial-initiation latency). We found that a large decrease in reward probabilities rapidly produced the greatest change in choice proportions. The time taken to execute a choice reflected the differences in reward probabilities across the two spouts in some cases, but also reflected training history. By contrast, the amount of time rats spent responding at reward spouts in anticipation of reward consistently reflected the relative likelihood of reward across the two spouts and not the absolute probability of reward. The latency to initiate the subsequent trial reflected choice evaluation. These three response latencies thus indexed key behavioral correlates of the choice process as it unfolds in time. We discuss how this paradigm can be used to assess the corresponding neural correlates of decision-making.

## Introduction

How do animals distribute their time and choices across options in order to maximize their procurement of food and other necessities? This is a key question in the context of foraging theory and adaptive decision-making. One fundamental feature of the environment within which animals make such choices is that the likelihood of resource availability changes over time. Hence, it is essential to study how choice behavior adjusts to changes in environmental conditions.

Another feature of natural environments is that information about the likelihood of reward is often not directly indicated and, hence, the value associated with one choice over others can only be obtained through the animal's exploration of alternative options. Animals need to first detect the change in outcome contingencies and then identify the corresponding ideal behavior. Such adjustment in choice behavior can be quantified in terms of the degree of adjustment, its optimality and the speed with which it occurs. Here, we trained rats in a discrete trial choice paradigm, and examined how specific aspects of unsignaled changes in choice outcome (the likelihood of a palatable sucrose solution) differentially impact on the speed and amount of changes in choice allocation. In addition we examined if behavioral latencies pre- and post-choice adjust to changes in the likelihood of the sucrose reward.

The impact of changes in reward contingencies on behavior has been examined in studies of contrast effects. The typical observation when animals are presented with two options is that a change in reward availability from one option results in a change in behavior toward the other unchanged option. A classic example involves first presenting pigeons with a red and green light in succession, where each light is associated with equal reward rates. Reynolds (1961) found that changing the reward rates associated with only one of the lights produced a change in response rate to the other light; an increase in response rate to the green light following a decrease in reward associated with the red light is termed positive contrast, while a decrease in response rate to green following an increase in reward associated with red is termed negative contrast. In conditions where reward contingencies are simultaneously available (red and green lights presented at the same time), contrast effects manifest as a ratio of choices to the lights which match the ratio of rewards obtained (the Matching Law, Herrnstein, 1961; Catania, 1973; Rachlin, 1973). These studies of contrast effects demonstrate that the relative, as opposed to absolute, rate of reward between two options exerts control over behavior and provides the framework with which we quantified how the rats in the present study respond to changes in reward contingencies.

In the current study, rats nose-poked to initiate a trial which consisted in two simultaneous options. These options were associated with reward contingencies that changed across three phases. Critically, the reward likelihood for one option remained unchanged, while the other option either increased or decreased by small or large amounts. We identified changes in choice allocation across the three phases, and further examined changes in pre- and post-choice response latencies. The discrete trial structure used here allowed us to decompose a single choice into three distinct components with which we could examine behavioral latencies as an index of choice behavior. Our previous work has shown that choice execution latencies (duration between leaving the nose-poke and first contact with a reward spout), spout sampling durations (time between first and last reward spout contact on unrewarded trials) and trial initiation latencies (duration between last spout contact and arrival at nose-poke) are sensitive to reward contingencies (Fam et al., 2015). In particular, we found spout sampling durations to be the most sensitive and consistent index of changes in reward probabilities (Lavan et al., 2011; Fam et al., 2015). Here, we used the same three measures to identify how changes in reward contingencies can drive changes in choice allocations and choice latencies. Specifically, we assessed if response latencies within these three distinct components of choice would track the changes in reward probabilities across the three phases, thereby extending our previous work which assessed response latencies with one change in reward contingencies.

We compared changes in behavior when reward probabilities on one alternative remained constant while the probabilities on the other increased or decreased by the same amount in different groups. This was done in order to assess whether responding to the unchanged spout was affected by the changes in reward probability on the other spout. Comparisons of behavior when reward probabilities increase versus decrease by an identical amount would be interesting. In both situations, one of the reward spouts (High) has a greater probability of reward compared to the other (Low); however, the absolute reward probabilities for High for one group would be identical to the reward probabilities for Low in another group. Contrast effects would be evident if behavior toward these two spouts is different (reflecting the different relative values). However, if behavior reflects absolute value, then behavior toward these two spouts will be identical (as a result of the identical absolute probabilities). The present study additionally examines these contrast effects across more than one change in reward contingency.

## Materials and methods

### Subjects

Subjects were 24 experimentally naïve, adult, male Wistar rats with initial weights of 159–191 g. Rats were housed in groups of four in opaque plastic boxes (22 cm high × 67 cm long × 40 cm wide) in a climate controlled colony room on a 12 h light-dark cycle, where lights were turned on at 7 a.m. All procedures were approved by the Animal Care and Ethics Committee at the University of New South Wales. Rats were given 1 h free access to food and water after each session and body weights were monitored to ensure they did not drop below 85% of their free-feeding weight.

### Apparatus and task

**Figure 1:**
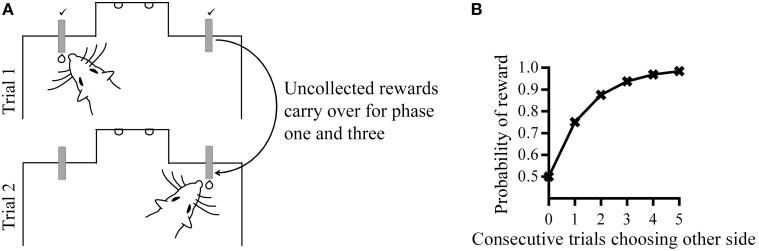
**(A)** Behavioral apparatus and task. **(B)** Reward hold contingencies during phase one and three; the probability of reward at one spout increases with consecutive trials choosing the other spout. The optimal choice strategy would be to alternate between the two spouts.

Rats were trained in a dark chamber (30 height × 20 width × 50 cm length) with a central nose-poke aperture (4 × 4 cm) located on the front wall of the chamber. Reward spouts were located at a distance of 5 cm on either side of the nose-poke aperture and delivered 5% sucrose solution via pumps located outside the testing chamber. Electrical sensors fitted to the reward spouts detected licking activity at a temporal precision of 1 ms. The experiment was controlled and data recorded using MATLAB (The MathWorks, Inc.). A schematic of the testing apparatus is shown in Figure 1A. Each trial started with a subject-initiated nose-poke which had to be maintained for a variable delay (nose-poke delay; 100–600 ms, uniform distribution). Two diode lights at the front wall of the nose-poke chamber were lit after this delay (go-signal). Rats then made a choice between the two spouts for rewards (0.5 s delivery of sucrose solution) delivered probabilistically and after another variable delay (reward delay; 100–600 ms, uniform distribution) throughout which rats had to maintain licking. If no reward was available for the chosen spout, nothing occurred and there was no constraint as to when rats could initiate the next trail. Background noise (approximately 70 db in volume) masked any extraneous sounds. Each experimental session consisted of at least 250 trials.

In phase one, there was a 50% probability of reward on each spout, and an uncollected reward from a previous trial remained available, but did not accumulate. This “holding” of reward meant that in order to obtain the maximum amount of rewards, rats had to alternate between reward spouts because the probability of reward at one reward spout increased with a choice of the alternative spout (Figure 1B). This reward “hold” manipulation thus prevented rats from developing a bias for one of the spouts which might obscure the effects of subsequent contingencies changes. During phase two, there was a change in the reward probability in one of the two spouts and the “hold” manipulation was removed. Therefore, the optimal behavior during this phase was choice of the reward spout with the higher probability of reward.

Rats were allocated to four experimental conditions (*n* = 6) according to the change in reward probabilities scheduled in phase two (Table 1). Changes in reward probabilities differed in size (30% versus 15%) or direction (increase or decrease). A positive direction of change refers to an increase in reward probabilities (+) and a negative direction of change refers to a decrease in reward probabilities (−). Note that a 30% decrease in reward probability produces a higher ratio of relative reward probabilities (50/20 = 2.5) compared to a 30% increase in reward probability (80/50 = 1.6).

**Table 1:** **Experimental Groups and Contingencies**.

**Group**	**Phase 1**	**Phase 2**	**Phase 3 (return to phase 1)**
+30	50–50 with reward hold	80–50 without reward hold	50–50 with reward hold
+15	50–50 with reward hold	65–50 without reward hold	50–50 with reward hold
−15	50–50 with reward hold	50–35 without reward hold	50–50 with reward hold
−30	50–50 with reward hold	50–20 without reward hold	50–50 with reward hold

At phase three, contingencies were identical to those in phase one. The aim of this three-phase manipulation was to first establish equivalent responding on both reward spouts in phase one against which the reward probability manipulations in phase two could be assessed, where we inquire how quickly rats adopt the new optimal strategy. At phase three, reward probabilities were returned to the initial contingencies to identify how quickly rats revert to the original optimal strategy of alternating. Rats were moved on to the next phase if and when they reached specific criteria (see Procedure) in an attempt to ensure that rats were at similar level of performance before moving on to the next phase. An additional constraint was that each phase had a maximum length of five sessions in the event that rats failed to reach criteria levels of performance. The choice of five sessions was based on our previous work where we found that rats made behavioral adjustments within 5 sessions following contingency reversal (Lavan et al., 2011; Fam et al., 2015). Constraining the session length was important for ensuring that rats would not have vastly different levels of experience with phase contingencies.

### Procedure

#### Spout shaping

Rats were placed in the experimental chamber for 10 min and sucrose was freely available from both reward spouts. The nose-poke aperture was blocked at this stage.

#### Nose-poke shaping

Rats were rewarded with sucrose only after performing a nose-poke. The nose-poke delay and reward delay were gradually increased over three shaping sessions (first session: 100 ms; second session: 100–400 ms; third session: 100–600 ms).

#### Training phase one

Rats were presented with 50–50 Hold reward probabilities for a maximum of five sessions, or until they reached criterion of alternating between reward spouts on more than 75% of trials in a session. At this stage, the experimental contingencies were identical for all rats.

#### Training phase two

Rats were presented with the reward probabilities that correspond to their experimental group identity (see Table 1). For all four groups, the reward probability for one of the spouts remained at 50%, while the other reward spout increased (+) or decreased (−) by 30% or 15%. Hence, while groups +30 and −30 experienced a change in reward probability of identical size (30%), this was an increase for group +30 (from 50 to 80%) but a decrease for group −30 (from 50 to 20%). Similarly, groups +15 and −15 both experienced a change in reward probability of 15%; this was an increase for group +15 (from 50 to 65%) but a decrease for group −15 (from 50 to 35%). Phase two again lasted for a maximum of five sessions or until rats reached criterion of choosing High on more than 80% of trials for a session.

#### Training phase three

This was identical to phase one.

### Analysis

Analyses were carried out using Graphpad, SPSS and MATLAB. Results are reported according to the spout status during phase two (High or Low). In addition to choice allocation, we focus on three response latency measures which our previous work has shown to be sensitive to reward contingencies (Fam et al., 2015). These were: choice execution latency (duration between leaving the nose-poke and first contact with a reward spout), spout sampling duration (time between first and last reward spout contact on unrewarded trials) and trial initiation latency (duration between last spout contact and arrival at nose-poke).

#### Quantifying changes in choice allocation

**Figure 2:**
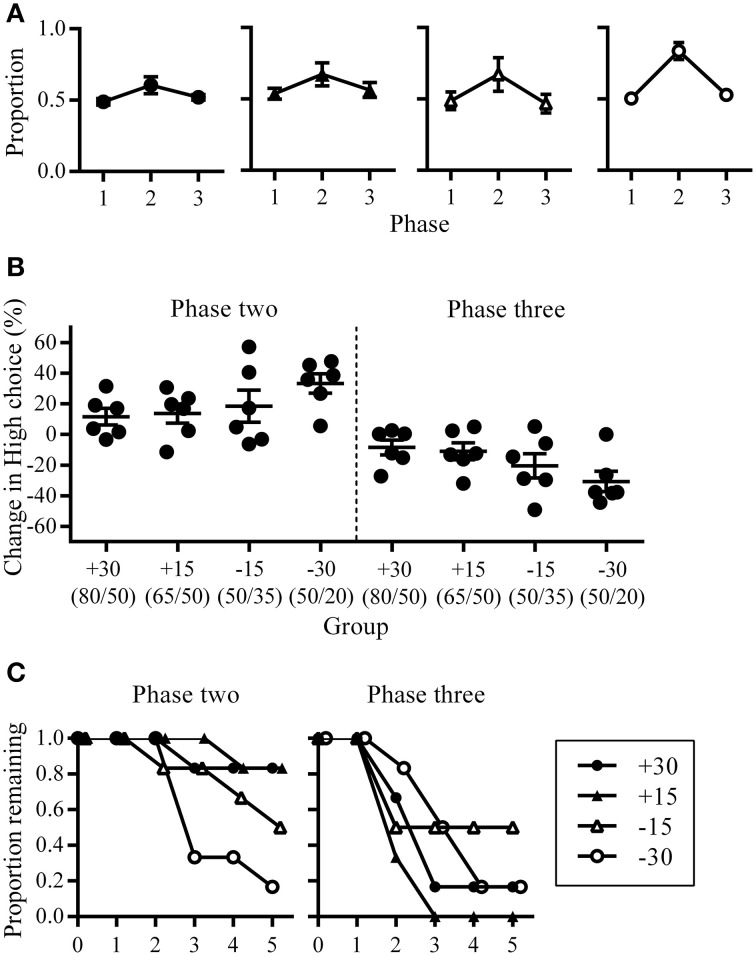
**Choice allocation between phases. (A)** Group mean proportion of High choice at the last session of each phase shown separately for each group. **(B)** The amount of change in High choice proportion at phase two (left) and three (right). This was calculated as the difference in High choice proportion between the last sessions of successive phases and expressed as a percentage. **(C)** Proportion of rats remaining at different phase lengths during phase two (left) and phase three (right).

**Figure 3:**
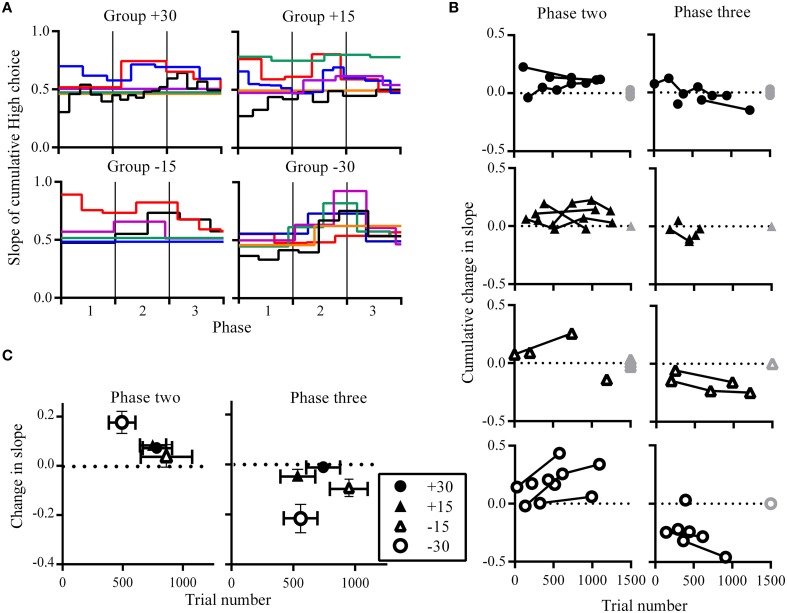
**Choice allocation within phases. (A)** Change-points and slope of the cumulative sum of choices made to High. Each colored line represents an individual rat and these are grouped according to experimental conditions. Step-like changes in the slope correspond to significant changes in the number of choices to High. Trial numbers are expressed as a proportion of phase length, enabling phase transitions across rats to be aligned. Black vertical lines indicate phase transitions. Change-points were not detected for some rats and these are shown as flat horizontal lines. **(B)** Cumulative change in the slope of the cumulative sum of High choices as they occurred across trials. Successive change-points from individual rats are linked. Gray symbols at the end of phases indicate rats for which no change-points were detected, where they have no change in slope (0 on the y-axis) at the maximum trial number (1500 on the x axis). These gray symbols are overlapping but are plotted with a slight jitter along the y-axis to indicate more than one data-point. **(C)** Group mean for the change in slope of the cumulative sum of High choices against the group mean for when they occurred in trial numbers. Vertical error bars indicate SEM for changes in slope, while horizontal error bars indicate SEM for trial number at which these changes occurred.

In addition to characterizing the changes in overall choice allocation between phases (Figure 2), we also assessed within-phase changes in choice allocation using a change-point algorithm (Gallistel et al., 2004). The cumulative sum of High choices across the entire experiment was examined to identity changes in the slope of the cumulative sum of High choices using a binomial probability test with decision criteria of logit value 2. The MATLAB functions and scripts for this algorithm were downloaded from the Proceedings of the National Academy of Science, USA website, which were made available by the authors (Gallistel et al., 2004). This change-point analysis allowed us to quantify the latency at which changes in choice proportions occurred (trial number) and the amount of change that occurred (slope of the cumulative sum) in response to changes in reward contingencies. To facilitate visualization of the step-like changes in the slope of the cumulative sum of High choices across rats that experienced different phase lengths due to the criterion-based design of this experiment, we expressed the trial numbers at which change points occurred as a proportion of phase length (Figure 3A). All other analyses with regards to the latency at which change-points occurred and the size of change observed were performed using raw (un-transformed) trial numbers. To further quantify the emergence of shifts in choice allocation toward optimality in response to phase contingencies, we examined the latency with which adaptive changes occurred and the degree of such changes. Because optimal performance at phase two was the exclusive choice of High, adaptive change points during phase two were defined as increases in High choices from phase one. At phase three, alternating between rewards spouts was the optimal strategy, and adaptive change-points here were defined as a decrease in High choices from phase two.

#### Quantifying changes in response latencies

The analysis of choice proportions revealed that changes could occur rapidly within one session. Therefore, to quantify the changes in behavioral latencies in greater detail across the three phases, we first divided individual sessions into bins of 50 trials. Latencies for each bin were calculated separately for High and Low. Each rat's High and Low bin latency was then expressed as a proportion of its own baseline (calculated as the median latency for the last session of the previous phase, where the median was calculated by combining both High and Low latencies). Hence, phase two latencies are expressed relative to phase one baseline, and phase three latencies are expressed relative to phase two baseline. Previous phase baseline was calculated using spout sampling durations for both High and Low as this represented the experience of the rats, where reward probabilities were equivalent across the two spouts at phase one and three. Group means for High and Low proportion latencies are shown in **Figure 5**. We also compared latencies in phase three against those in phase one to assess whether choice behavior was the same between these two phases, reflecting the identical reward contingencies (**Figure 6**). Supplementary figure 5 shows the three latency measures not relative to baseline.

To determine whether the difference between High and Low proportion latencies were statistically significant within groups, we carried out a randomization test. High and Low proportion latencies were combined and shuffled. The mean of the first half of this shuffled sample was then subtracted from the mean of the second half of the shuffled sample. This was repeated 1000 times to generate a distribution of the difference in proportion latencies when spout identity (High versus Low) was disregarded. This test identifies the differences in proportion latencies which arise specifically from the differences in spout identity without making assumptions about the underlying distribution of proportion latencies. This randomization test was carried out separately for each group. Significance was determined by comparing the observed difference in group means with the 5th–95th percentile of the randomized distribution and calculating two-tailed *p*-values. When comparing between trial initiation intervals after different trial outcomes at the same reward spout, randomized distributions were computed based on trial outcome (rewarded versus unrewarded). We also calculated the effect sizes of the differences between latencies using Cohen's *d* (Cohen, 1988) in order to make between-group comparisons. Note that **Figures 5**, **6** show the mean group latencies for High and Low separately, and not the group mean difference (Low subtracted from High) which was used to determine significance using the randomization test. We plot group mean latencies separately for High and Low in **Figures 5**, **6** because this enables visualization of the source of the differences in means (increase in High from baseline versus decrease in Low from baseline), which is not captured by plotting the group mean difference in latencies.

## Results

### Choice allocation between phases

Following the change in High/Low reward ratios at phase two, all groups increased choices to High. Group −30 showed the largest increase in both High choice (Figure 2A) and the percentage change in High choice proportions across the three phases (Figure 2B), while all other groups were similar. Minus groups had the greatest number of rats who reached criterion at phase two (group +30: *n* = 1; group +15: *n* = 1; group −15: *n* = 3; group −30: *n* = 5; Figure 2C), while at phase three, group −15 had the smallest number of rats who reverted back to an alternating strategy within five sessions (group +30: *n* = 5; group +15: *n* = 6; group −15: *n* = 3; group −30: *n* = 5; Figure 2C).

### Changes in choice allocation within phases

The pattern of changes in choice allocation between phases was further examined for each rat within phases two and three. Figure 2 shows that changes in choice proportions mirrored the changes in reward probabilities; the slope of cumulative High choice increased during phase two and decreased during phase three for most rats (Figure 3A). This pattern was robust overall although change-points at this decision criteria (logit of 2) were not detected for some rats (group +30: *n* = 3; group +15: *n* = 1; group −15: *n* = 3; flat horizontal lines in Figure 3A). It is worth noting that change-points were detected for all rats in Group −30 when the analysis was repeated with the highly conservative criteria of logit 4. Across phases two and three, group −30 showed both the shortest latency to make adaptive changes to choice allocation, and the greatest amount of change in choice allocation (Figures 3B,C), with all other groups being similar. The similarity among the other groups indicates that neither size nor direction of change in reward contingencies alone determined the adjustment of choice proportions. Rather it was the joint contribution of the large and the negative change in reward availability that facilitated choice adjustment in Group −30.

### Temporal components of choice behavior

**Figure 4:**
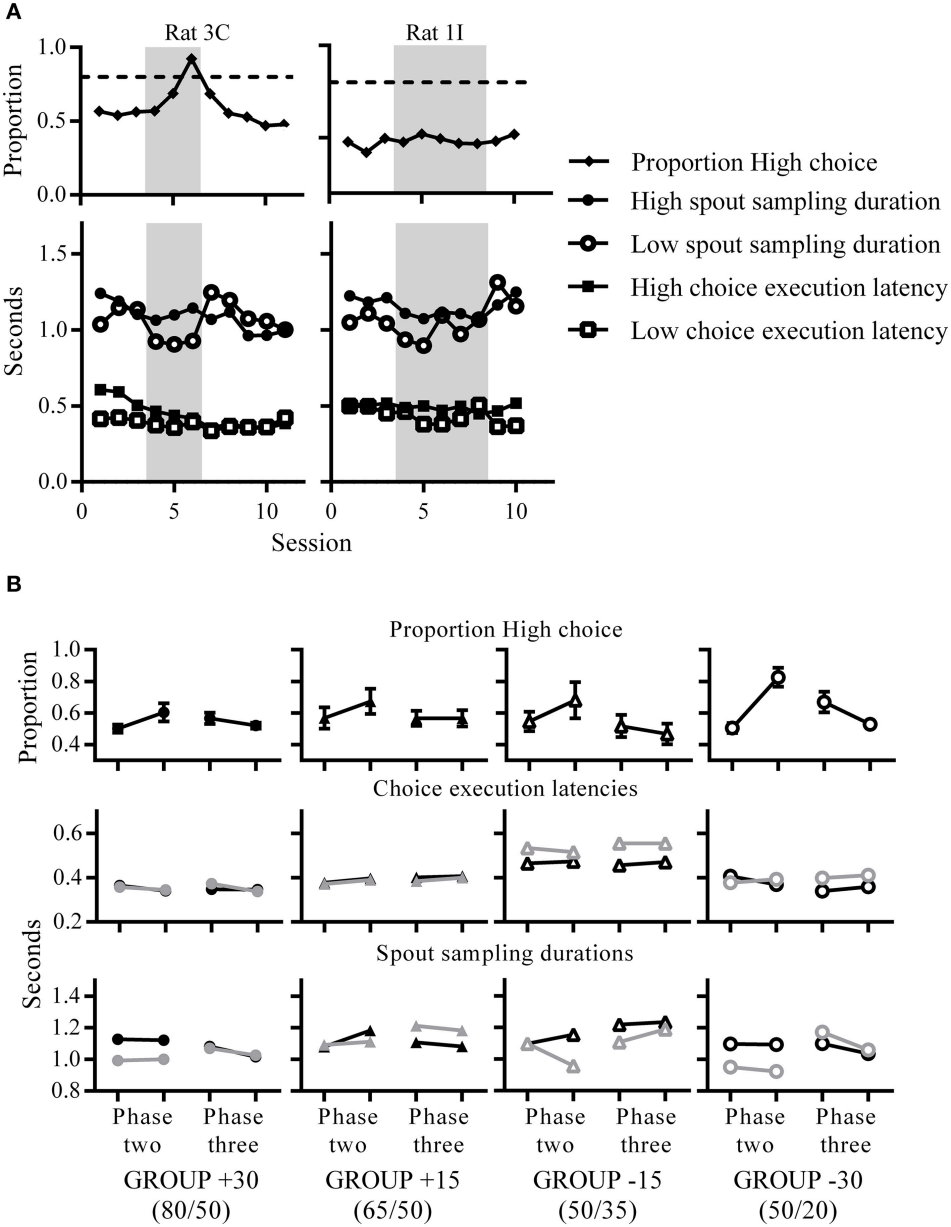
**Changes in choice proportions relate to changes in latencies. (A)** Choice proportions and median choice execution latencies and spout sampling durations across sessions. An example rat that was choosing High almost exclusively (left) and another example rat which was choosing both spouts equivalently (right) at phase two (gray shaded portions indicate phase two contingencies). Both rats showed longer spout sampling durations for High compared to Low during phase two. **(B)** Group mean High choice proportions (top), and group median choice execution latency (middle) and spout sampling durations (bottom) for the first (left data point) and last (right data point) session of each phase. For the middle and bottom panels, black symbols indicate medians for High and gray symbols indicate medians for Low.

Since the changes in reward probabilities were reflected in changes in choice proportions, we sought to identify similar changes in other indices of choice behavior. A striking pattern was that spout sampling durations were longer for High than Low during phase two only, reflecting the change in relative reward probabilities between the two spouts across phases (Figures 4A,B). This was evident for rats that chose High almost exclusively but also for rats that distributed their choices equally between the two spouts. Figure 4A shows an example of a rat exhibiting exclusive choice and of one that distributed its choices. Although, some rats did not discriminate between High and Low reward spouts in terms of choice proportions, these rats did modulate the amount of time spent waiting for rewards on unrewarded trials in a manner consistent with reward contingencies (Figure 4B and Supplementary figures 1–4). This indicates that time spent at the spout, an index of post-choice outcome expectancy, is more sensitive than overt choice in reflecting environmental contingencies. This difference in spout sampling durations was also clear in group medians, as all groups showed longer spout sampling durations at phase two for High than Low; a difference that was present after a single session of phase two contingencies in the two groups subjected to the largest change (+30, and −30; Figure 4B). Following the decrease in reward ratios at phase three, rats adjusted their spout sampling durations to reflect the equivalence between reward probabilities at the two spouts and by the end of phase three, groups +30, −30 and −15 (but not +15) were sampling both spouts for equivalent durations on unrewarded trials.

### Pre-choice behavior: Choice execution latencies

**Figure 5:**
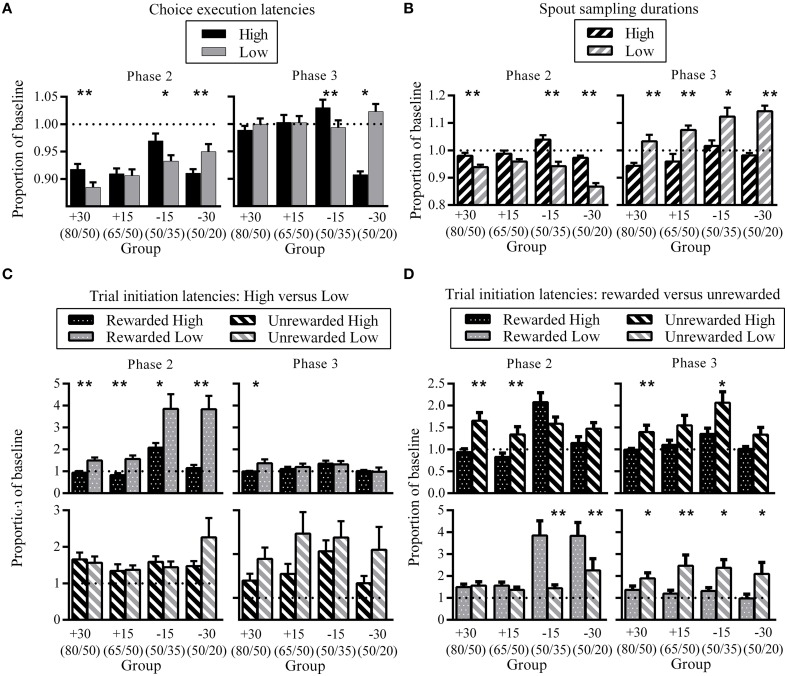
**Differences in choice latencies between High and Low. (A)** Group mean High and Low choice execution latencies as a proportion of previous phase baseline at phase two (left) and phase three (right). **(B)** Group mean High and Low spout sampling durations for unrewarded trials as a proportion of previous phase baseline at phase two (left) and phase three (right). **(C)** A comparison of High versus Low trial initiation latencies for identical trial outcomes as a proportion of previous phase baseline at phase two (left) and phase three (right). Top panels show group mean trial initiation latencies after High and Low rewarded trials, bottom panels show group mean trial initiation latencies after High and Low unrewarded trials. **(D)** Effect of trial status on trial initiation latencies for the same reward spout as a proportion of previous phase baseline at phase two (left) and phase three (right). Top panels show group mean trial initiation latencies after rewarded and unrewarded trials at High, bottom panels show group mean trial initiation latencies after rewarded and unrewarded trials at Low. All error bars indicate SEM, and the dashed horizontal line indicates where data would lie if there was no difference from baseline. ^*^p < 0.05; ^**^p ≤ 0.01.

In order to quantify changes in behavioral latencies that result from contingency changes, we calculated the amount of change by each rat from its baseline (see Materials and Methods). Figure 5A shows choice execution latencies for each group at phase two (left) and phase three (right) as a proportion of baseline. At phase two, choice execution latencies for all groups were shorter than baseline for both High and Low spouts, which reflects an increased execution speed as a result of training. Across both phases, there was a tendency for High choice execution latencies to be greater than Low, although latency differences in group −30 showed the opposite direction. The difference between latencies to High and Low was significant for all groups except group +15 at phase two, and groups +30 and +15 at phase three. Effect sizes reveal that at phase two, groups which experienced the larger change of 30% showed the greatest High-Low differences (group +30: *d* = 0.42; group −15: *d* = 0.20; group −30: *d* = 0.25), while phase three effect sizes were greatest for group −15 (group −15: *d* = 0.30; group −30: *d* = 0.21).

### Post-choice behavior: Spout sampling durations

In phase two, spout sampling durations were significantly longer on the High than the Low side, except for group +15 where the difference approached but did not reach the conventional level of significance (Figure 5B; group +15: *p* = 0.06). Effect sizes indicate that these differential spout sampling durations were greatest for groups −15 and −30 (group +30: *d* = 0.27; group −15: *d* = 0.41; group −30: *d* = 0.68). The comparison of spout sampling durations with baseline also show that the differences in spout sampling durations were due to a decrease in the Low spout sampling durations from phase one rather than an increase in High spout sampling durations: Low proportion latencies were all less than 1, while High proportion latencies were close to 1 (Figure 5B). This decrease in spout sampling duration mirrors the decrease in reward probabilities for −15 and −30 groups, and also the decrease in the relative value of the Low side for +15 and +30 groups as a result of the increase on the High side.

In phase three, consistent differential sampling times were again apparent (Figure 5B, right). Here, spout sampling durations were significantly greater on the Low than on the High side relative to phase two baseline for all groups, and effect sizes indicate that the greatest difference was for group −30 (group +30: *d* = 0.44; group +15: *d* = 0.48; group −15: *d* = 0.28; group −30: *d* = 0.75). These differences were due to an increase in spout sampling durations on Low, while High spout sampling durations remained close to baseline (Low proportion latencies all greater than 1). These changes again reflected the current contingencies in that the increase in spout sampling time on the Low side mirrors the increase in reward probabilities for −15 and −30 groups and the increase in the relative value of the Low side for +15 and +30 groups.

### Post-choice behavior: Trial initiation latencies

To assess changes in trial initiation latencies, we analyzed data based on the four different trial outcomes of rewarded or unrewarded on the High and Low sides. A comparison of the effect of spout status (High versus Low) for identical trial types at phase two showed that all groups were significantly slower to initiate a new trial subsequent to receiving a reward from Low compared to one from High (Figure 5C, top left). Effect sizes indicate that group −30 showed the greatest differences in proportion latencies (group +30: *d* = 0.30; group +15: *d* = 0.33; group −15: *d* = 0.22; group −30: *d* = 0.45). There were no latency differences to initiate a new trial following unrewarded trials (Figure 5C, bottom left). In phase three (Figure 5C, right), differential latencies after rewarded trials (Figure 5C, top right) were only evident for group +30 (longer proportion latencies for Low), and there were no significant differences following unrewarded trials (Figure 5C, bottom right).

Figure 5D shows the effect of trial outcome (rewarded versus unrewarded) for the same spout location. A comparison of trial outcome on the High side in phase two (Figure 5D; top left) showed that groups +30 and +15 took significantly longer to initiate a new trial after unrewarded trials on High compared to rewarded trials (group +30: *d* = 0.29; group +15: *d* = 0.22). The corresponding comparison on the Low spout (Figure 5D; bottom left) indicated no such pattern. Groups −15 and −30 showed the opposite pattern; there were no differences between trial outcomes for trial initiation latencies on the High side (Figure 5D; top left), while latencies to initiate a new trial were significantly longer on the Low side after a rewarded than an unrewarded choice (Figure 5D, bottom left). Effect sizes show that the greatest difference was for group −30 (group −15: *d* = 0.32; group −30: *d* = 0.38).

In phase three (Figure 5D, right), latencies were longer after unrewarded than rewarded trials for both High (Figure 5D, top right) and Low (Figure 5D, bottom right) reward spouts. For the High reward spout, the difference between rewarded and unrewarded trials was only significant for group +30 (*d* = 0.3), while for the Low reward spout comparison between rewarded and unrewarded choices were significant for all groups except +30; effect size for group +15 was largest (group +15: *d* = 0.56; group −15: *d* = 0.3; group −30: *d* = 0.26).

### Phase three latencies compared to phase one latencies

There were inconsistent patterns in the difference between High and Low choice execution latencies during phase three expressed as a proportion of phase one baseline. While some groups showed longer latencies for choice execution for High compared to Low (group +30: *d* = 0.56; group −15: *d* = 0.24), groups +15 and −30 showed the opposite pattern (not significant for group +15; group −30: *d* = 0.24). This may reflect individual differences between rats as to the temporal location of the decision point. For some rats, the decision as to which spout to respond next might have been formulated prior to nose-poke entry, while other rats might have come to such a decision upon seeing the go-signal.

Spout sampling durations in phase three were similar to phase one baseline for groups +30, +15, and −30 while group −15 showed increased spout sampling durations relative to phase one. Critically, differential spout sampling durations between High and Low were no longer evident.

**Figure 6:**
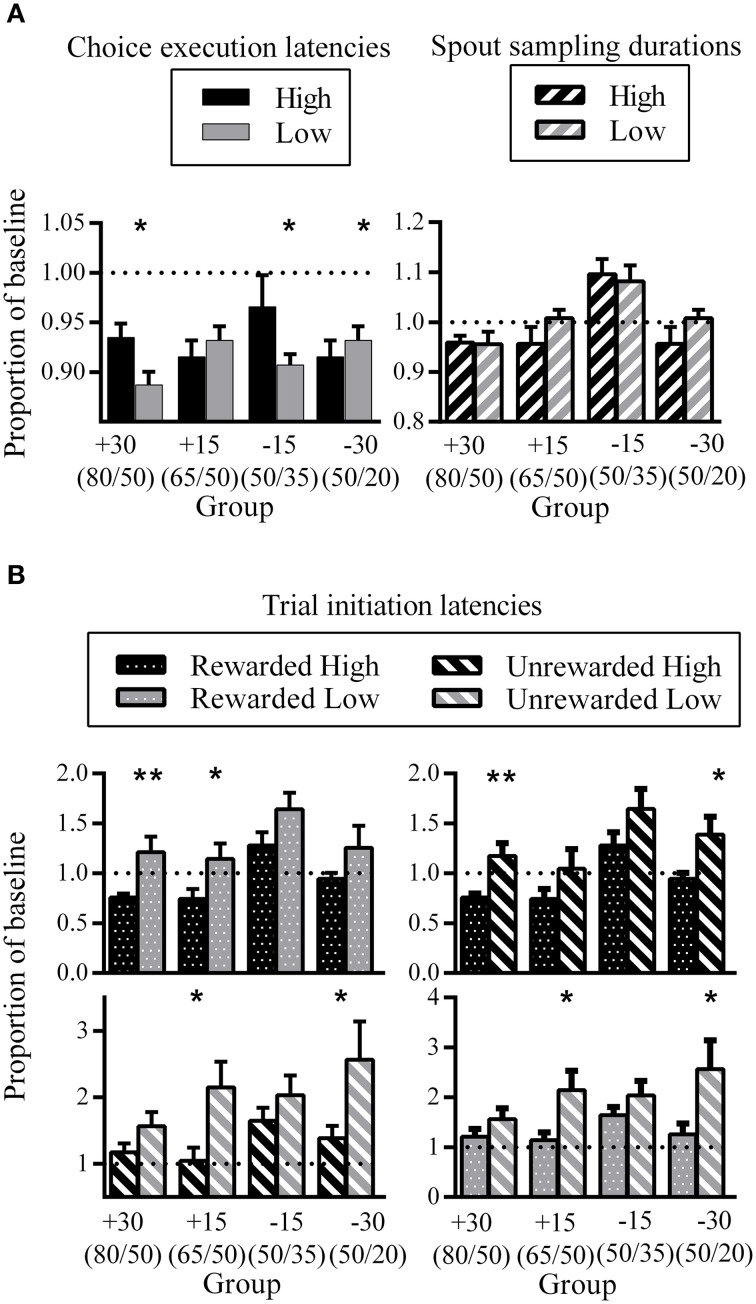
**Phase three latencies as a proportion of phase one baseline. (A)** Group mean choice execution latencies (left) and spout sampling durations (right). **(B)** Group mean trial initiation latencies following different trial outcomes. All error bars indicate SEM, and the dashed horizontal line indicates where data would lie if there was no difference from baseline. ^*^p < 0.05; ^**^p ≤ 0.01.

The comparison of trial initiation latencies for the same outcome but different spout showed that all groups took longer to initiate a trial after having visited the Low side, regardless of outcome (Figure 6B, top and bottom left). This was significant for groups +30 and +15 for the comparison between rewarded trials (group +30: *d* = 0.33; group +15: *d* = 0.28) and for groups +15 and −30 for the comparison between unrewarded trials (group +15: *d* = 0.34; group −30: *d* = 0.19). The corresponding comparison of different trial outcomes for the same spout showed that all groups took longer to initiate a new trial after unrewarded trials. This was significant for groups +30 and −30 for the High spout comparison (group +30: *d* = 0.35; group −30: *d* = 0.22) and for groups +15 and −30 for the Low spout comparison (group +15: *d* = 0.30; group −30: *d* = 0.28).

## Discussion

This experiment examined changes in choice allocation and choice latencies in response to changes in the contingencies between a location and reward. Rats chose between two spouts which delivered a sucrose reward probabilistically. Across three phases, we systematically manipulated the size as well as the direction of changes in reward probabilities in order to quantify how specific changes in environmental contingencies affect behavior. The aim of this three-phase manipulation was to first establish equivalent responding on both reward spouts (phase one) against which the reward probability manipulations in phase two could be assessed, where we inquired how quickly rats adopted the new optimal strategy. In phase two, different groups were exposed to a large or small increase or decrease in reward probability associated with one of the reward spouts, while probability of reward on the other spout remained unchanged at 50% for the entire experiment. In phase three, reward probabilities were returned to the initial phase one contingencies to identify how quickly rats reverted to the original optimal strategy of alternating. In addition to identifying the adjustment in choice allocation in response to phase contingencies, we examined adjustments in the latencies of pre- and post-choice behaviors. These latencies mirrored changes in choice allocation, but also reflected other aspects of the choice process not captured by choice allocation.

The most direct measure of perceived changes in reward probability at phase two was choice allocation. In terms of criterion performance, group −30 appeared to be the most sensitive, with half of the group reaching criterion within the third session and all but one rat reaching criterion at the end of five sessions. The corresponding size of reward probability change experienced by group +30 did not result in comparable criterion performance. A change-point algorithm (Gallistel et al., 2004) further showed that most rats that did not reach choice criterion, had in fact made adaptive changes to their choice allocations by increasing choices to High in phase two, and decreasing choices to High in phase three. The change-point algorithm also enabled a quantification of how quickly choice adapted and the size of the change in choice when reward contingencies changed. Across the various between- and within-session measures of changes in choice allocation in phase two, the large decrease in reward probabilities experienced by group −30 elicited the quickest and largest adaptations in choice allocation. The increase in reward probabilities experienced by group +30 did not result in comparable levels of change in behavior. This might indicate that the effects of a decrease in reward probabilities are not symmetrical to those produced by an increase in these reward probabilities. This suggestion is supported by between-group comparisons of effect sizes across the various response latencies. Overall, group −30 showed the greatest difference between latencies across phases two and three, while group +15 showed the smallest degree of change. This pattern of latency measures suggests that the direction (increase versus decrease) of the changes in reward probability exerts a larger effect on changes in response latencies between High and Low spouts, while the size of the changes in reward probability results in smaller effects. An alternative explanation is that phase two contingencies for groups +30 and +15 are more similar to phase one contingencies compared to groups −15 and −30. As seen in Figure 1B, if rats were alternating between reward spouts at phase one, the experienced reward probabilities would have been close to 75% on average for each session. Thus, the subsequent increase in reward probabilities to 80% on High during phase two for group +30 would have been harder to detect compared to the decrease in reward probabilities for group −30. This could account for our observation that group +30 did not show as much change in choice behavior as group −30.

Asymmetrical effects of increases versus decreases of reward probabilities were also apparent in spout sampling durations. These durations can be an index of choice confidence and our previous work has found this to be a robust and sensitive measure of choice preference (Lavan et al., 2011; Fam et al., 2015). Here, we found that changes in spout sampling durations between High and Low tracked the changes in reward probabilities across the three phases. There were two key findings. Firstly, spout sampling durations were longer for High compared to Low across all groups during phase two. This greater persistence in responding for High compared to Low reflects the greater likelihood of reward from High. Secondly, differential sampling durations were generated by changes in behavior to Low, not to High. In phase two, rather than increasing the duration of spout sampling for High, rats decreased this duration for Low. This was observed across all groups, regardless of whether the difference in reward probabilities between High and Low was due to an increase on one spout (as for +30 and +15 groups) or a decrease (−30 and −15 groups) on the other. Similarly, in phase three, all groups increased spout sampling durations to Low, rather than adjusting the durations for High. These results show that the value assigned to a choice is determined by its relation to other available choices in the environment, rather than the objective contingencies associated with that choice in isolation. If choice behavior was determined by absolute reward probabilities, we would have observed identical spout sampling durations for Low for + groups, as to High for—groups during phase two. Another implication of the differential spout sampling durations observed here is that the scale of choice behavior anchors to the most valuable option, hence in the current experiment, spout sampling durations for the High spout determined the maximum latencies or durations against which latencies to the Low spout were decreased in phase two or increased in phase three.

The pattern of differential spout sampling durations observed here are mostly consistent with the contrast effects reported in earlier studies (Reynolds, 1961; Rachlin, 1973; McSweeney and Norman, 1979). We observed negative contrast for groups +30 and +15 as indexed by decreased spout sampling durations to Low due to the increase in probabilities for High at phase two. However, we did not observe positive contrast for groups −30 and −15 at phase two, as would have been evidenced by increased spout sampling durations on High due to the decrease in probabilities for Low. This might be due to a ceiling effect imposed by the variable reward delay used; ceiling effects have been demonstrated to obscure positive contrast (Flaherty, 1982). Nevertheless, the present findings emphasize that choice behavior is controlled by the value of one alternative relative to other options. Importantly, spout sampling durations to the unchanged reward spout varied as a consequence of the reward probabilities, which were dynamic across the three phases. One way in which these dynamic changes can be further examined is via the use of electrophysiological recordings to identify any differences in neural activity to High versus Low that precede differences in spout sampling durations. Neurophysiological studies have shown that the firing rates of neurons in orbitofrontal cortex are mediated by relative value (Tremblay and Schultz, 1999; Padoa-Schioppa, 2009; Kobayashi et al., 2010; Rangel and Clithero, 2012). This might be a neuronal mechanism which underpins behavioral contrast. Another potential brain region, the amygdala, has been shown to directly mediate negative behavioral contrast (Henke et al., 1972; Becker et al., 1984). The amygdala, while well-known to be critical for fear learning, has recently been shown to be important for processing of reward-related information as well (Salinas et al., 1996; Baxter and Murray, 2002; Ambroggi et al., 2008).

The examination of trial initiation latencies in phase two also revealed that outcomes that were the least likely (unrewarded trials on High for +30 and +15 groups; rewarded trials on Low for −30 and −15 groups) led to longer trial initiation latencies. This window between trial outcome and initiation of a new choice might be a period of choice evaluation, when the immediate outcome is integrated with existing representations of environmental contingencies to guide upcoming choices. Hence, following surprising (unlikely) events, longer trial initiation latencies may reflect the updating of choice-outcome associations. Further, the differences in trial initiation latencies between the two outcomes were only evident for the spout which had been associated with new contingencies in phase two (High for + groups and Low for − groups). This is consistent with the suggestion that these latencies reflect choice evaluation, as outcomes from the unchanged spout were expected and did not require updating.

It is interesting that we observed differences in behavior between the changed versus unchanged spout in this choice evaluation period, when spout sampling durations instead reflected the High-Low difference in spout status. It is unlikely that the differences in trial initiation latencies between the two different outcomes simply reflect the time taken for reward consumption for two reasons. Firstly, as noted, the differences in trial initiation latencies between rewarded and unrewarded trials were only evident for one of the spouts; reward consumption effects would be evident for both spouts. Secondly, trial initiation latencies after rewarded trials at Low were longer than trial initiation latencies after rewarded trials at High. Since the reward was identical in size and concentration between the two spouts, the differences in latencies cannot be due to differences in the times taken to consume the sucrose.

The level of surprise associated with choice outcomes is a critical aspect of choice evaluation. Models of decision-making emphasize that learning to make adaptive choices is mediated by the ability to adjust our predictions of choice outcomes in line with experienced choice outcomes (Sutton and Barto, 1998; Bogacz, 2007; Sakai and Fukai, 2008; Stephens, 2008). This adjustment of the discrepancy between expected and actual outcomes, or prediction error, is proportional to the size of this discrepancy (Rescorla and Wagner, 1972; Dayan and Abbott, 2001). The trial initiation latencies here may represent the updating of predictions about the environment. In actor-critic models of decision-making, the basal ganglia has been suggested as the locus of choice-evaluation processes (O'Reilly and Frank, 2006; Prescott et al., 2006). In particular, the ventral striatum has been implicated to be involved in choice outcome evaluation (Cardinal et al., 2002; Nicola, 2007). Electrophysiological recordings while rats perform the current task could elucidate the role of various brain regions implicated in choice evaluation processes.

The present results show that choice allocation is sensitive to both aspects of the changes in reward probabilities manipulated here. We found asymmetrical effects of increases and decreases in reward probabilities on choice allocation and choice response latencies. Instead, choice behavior corresponded to the relative ratio of reward probabilities, where higher ratios had greatest effects on behavior. This emphasizes that relative rather than absolute reward rates guide behavior. Further, our results identified that High-Low differences in post-choice confidence, as indexed by spout sampling durations, were a product of decreased durations for Low. This was observed across all groups at phase two, despite the different directions of reward probability changes across groups. This study also found that changes differential spout sampling durations reflected the High-Low spout status, while trial initiation latencies reflected whether the reward probabilities for a spout had been changed versus unchanged. This also indicates that the three latency measures index different stages of choice as they unfold across the trial. The effects on choice allocation were also distinct from the effects on latency variables, showing that choice is a multi-stage process with distinct components within which various aspects of the environment have different effects.

Our findings with regards to response latencies indicate that the pre-choice (choice execution latency) and post-choice discrete periods (spout sampling duration and trial initiation latencies) index the different stages of decision-making. This is an ideal way in which the role of brain regions which have been argued to be critical for certain aspects of decision-making can be assessed. For example, studies have argued that the ventral striatum is important for choice outcome evaluation (Cardinal et al., 2002; Nicola, 2007), while the dorsal striatum is important for action selection (Balleine et al., 2007); this can be tested with online electrophysiological recordings while rats perform the present task. Similarly, the role of the amygdala in behavioral contrast could be examined by comparing neural activity for the different experimental groups, where neural activity for groups +30 and +15 might be different to that of groups −15 and −30 during phase two. Importantly, such neural recordings would allow a trial-by-trial readout of how neural activity relates to response latencies as well as choice allocation, thus identifying neural correlates which may precede changes in response latencies, and the way in which these guide choice allocation.

### Conflict of interest statement

The authors declare that the research was conducted in the absence of any commercial or financial relationships that could be construed as a potential conflict of interest.
