# Supplementary material for: Behavioral correlates of the decision process in a dynamic environment: post-choice latencies reflect relative value and choice evaluation
Source: Front Behav Neurosci. 2015 Sep 29;9:261. doi: 10.3389/fnbeh.2015.00261 (PMC4586275; doi:10.3389/fnbeh.2015.00261)
Supplement: Supplementary file 1 [file Image1.PDF]

GROUP +30

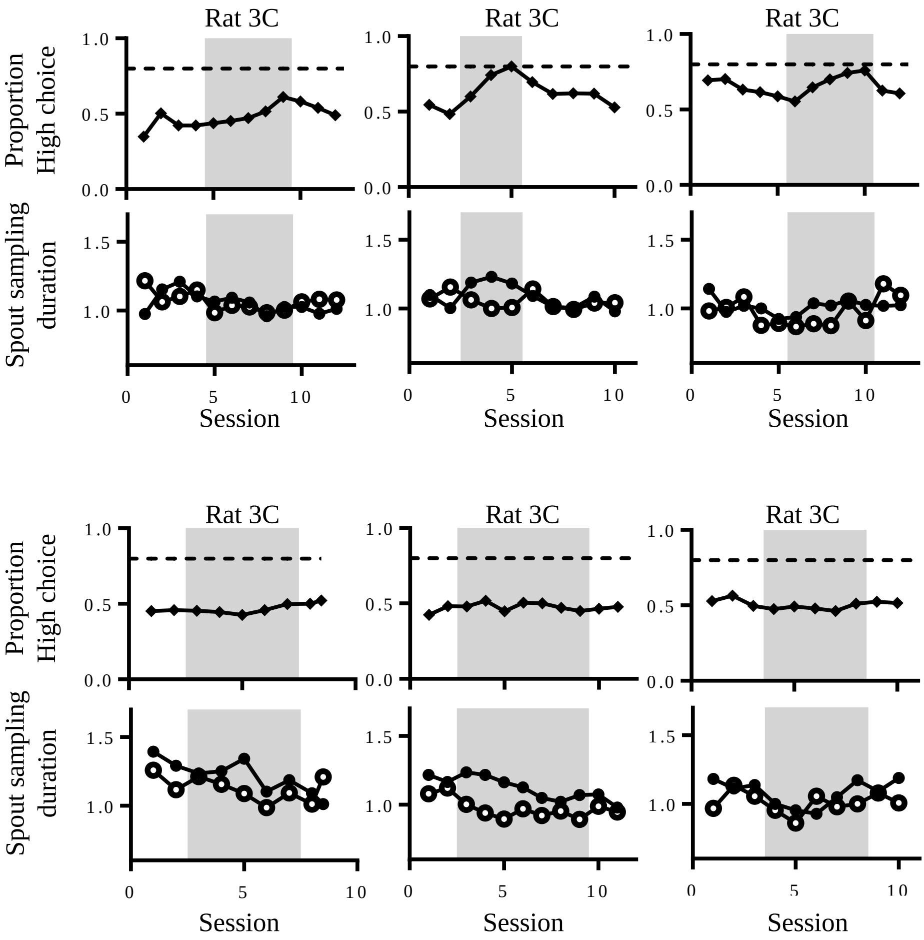

Supplementary figure 1. Choice allocation and median unrewarded spout sampling durations for individual rats in Group +30. Spout sampling durations are shown separately for High (filled circles) and Low (unfilled circles). Dashed horizontal lines indicate criterion for Phase two. Shaded grey portions indicate when Phase two contingencies were presented.
