# Supplementary material for: Behavioral correlates of the decision process in a dynamic environment: post-choice latencies reflect relative value and choice evaluation
Source: Front Behav Neurosci. 2015 Sep 29;9:261. doi: 10.3389/fnbeh.2015.00261 (PMC4586275; doi:10.3389/fnbeh.2015.00261)
Supplement: Supplementary file 2 [file Image2.PDF]

GROUP +15

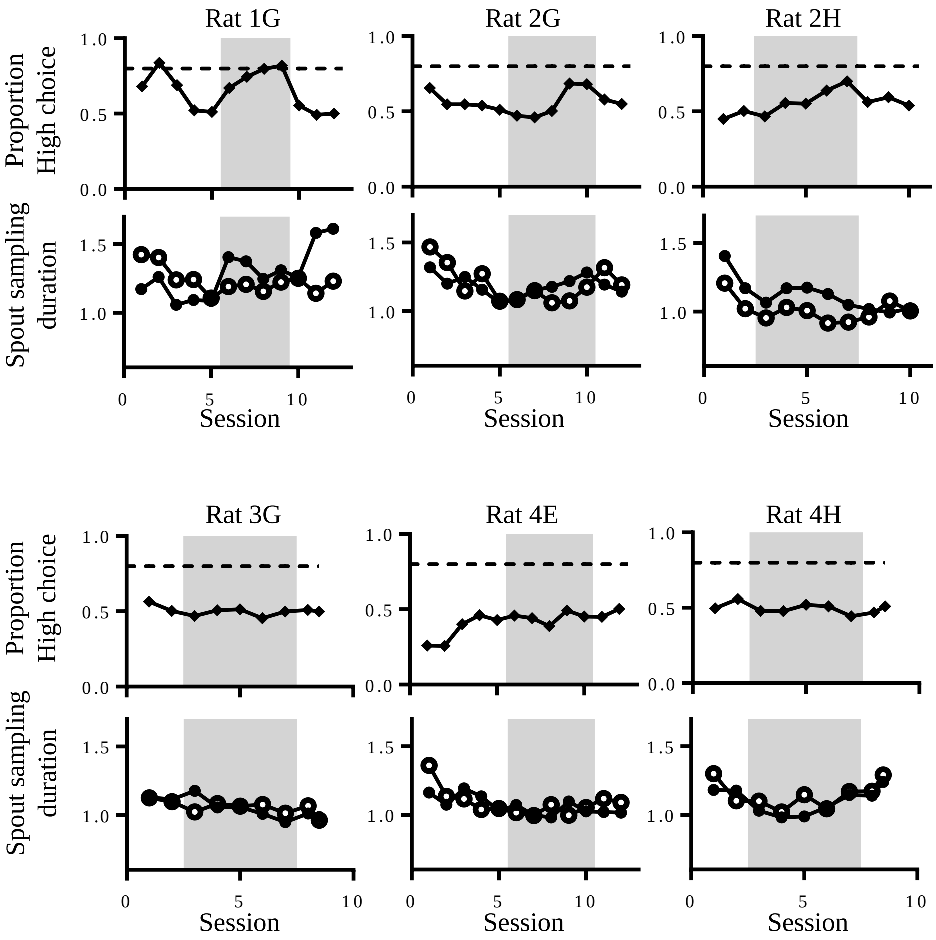

Supplementary figure 2. Choice allocation and median unrewarded spout sampling durations for individual rats in Group +15. Plotting conventions are as in Supplementary figure 1.
