# Supplementary figures and images for: Behavioral correlates of the decision process in a dynamic environment: post-choice latencies reflect relative value and choice evaluation
Source: Front Behav Neurosci. 2015 Sep 29;9:261. doi: 10.3389/fnbeh.2015.00261 (PMC4586275; doi:10.3389/fnbeh.2015.00261)

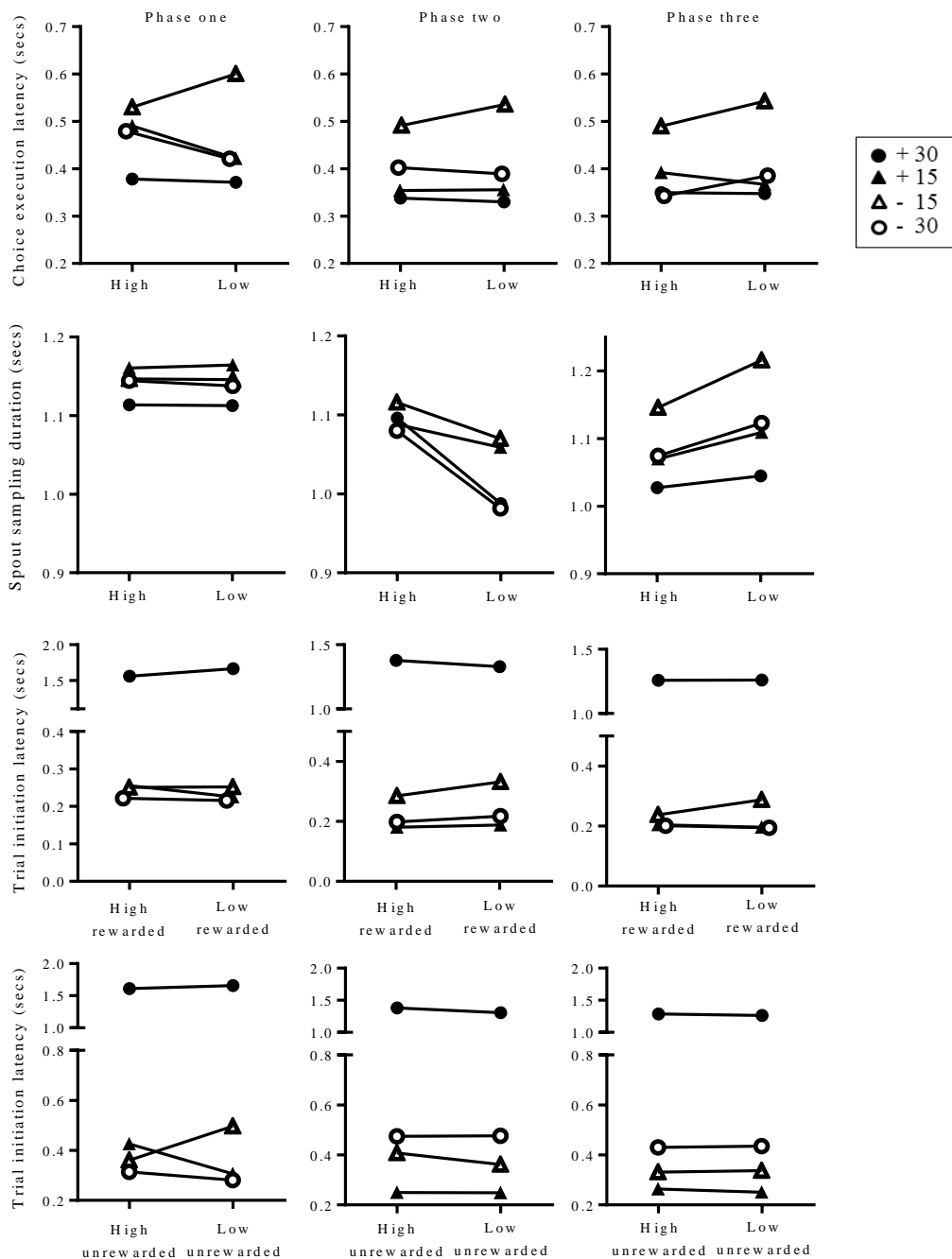

Supplementary figure 5. Median response latencies (not relative to baseline) for each phase.

Supplement: Supplementary file 5 [file Image5.PDF]
